# Effect of bioactive glass nanoparticles on biological properties of PLGA/collagen scaffold

**DOI:** 10.1007/s40204-018-0089-y

**Published:** 2018-05-11

**Authors:** Samira Nokhasteh, Alireza Sadeghi-avalshahr, Amir Mahdi Molavi, Mohammad Khorsand-Ghayeni, Hojjat Naderi-Meshkin

**Affiliations:** 1 Department of Materials Research, Iranian Academic Center for Education, Culture and Research (ACECR), Mashhad Branch, Mashhad, 91775-1376 Iran; 2 Stem Cell and Regenerative Medicine Research Department, Iranian Academic Center for Education, Culture and Research (ACECR), Mashhad Branch, Mashhad, 91775-1376 Iran

**Keywords:** 64S bioactive glass, PLGA/collagen, Ionic release, Skin substitute

## Abstract

Bioactive glasses have shown some interesting biological properties such as biocompatibility, biodegradation, and angiogenesis in skin tissue engineering. In the current research, the effects of MgO- or CoO-doped 64S bioactive glass with a composition of 64 SiO_2_-26 CaO-5 P_2_O_5_-5 MgO or CoO (mol%) were studied in relation with biological properties of electrospun [poly(lactic-*co*-glycolic acid) (PLGA)/collagen]. PLGA/collagen samples were rinsed in suspension of bioactive glass nanoparticles in distilled water with a concentration of 0.1 w/v and then freeze dried. Cell adhesion, viability, angiogenesis, and ionic release were performed and tested in culture medium containing fibroblast cells. Attachment and viability of fibroblast cells were increased significantly in bioglass-coated samples, while shrinkage in PLGA/collagen scaffold was reduced by the addition of bioactive glass. Vascular endothelial growth factor secretion in coated scaffold was dropped compared to the uncoated samples. This could be attributed to the fast degradation of glass nanoparticles, according to the inductively coupled plasma-atomic emission spectroscopy results.

**Electronic supplementary material:**

The online version of this article (10.1007/s40204-018-0089-y) contains supplementary material, which is available to authorized users.

## Introduction

Since the introduction of bioactive glasses in 1969, these materials have attracted a wide attention for tissue engineering applications. Bioactive glass (BG) or simply bioglass is a non-crystalline solid material, which consists of various oxides. Each oxide plays a specific role in the glass structure, and depending on the glass formation ability, they are divided into three categories: glass or network forming site, network modifier, and intermediate oxide. To degrade in contact with body fluids, the composition of BG is normally selected in a way which provides a poor chemical strength (Rahaman et al. 2011). During glass degradation, some events such as exchange and release of ions, formation of gel-like film on the glass surface, accumulation of ions, and nucleation of apatite crystals on the surface provide conditions for osteo-conduction as well as osteo-induction (Gerhardt and Boccaccini 2010). Although most research of bioactive glasses has been performed on hard tissues, these materials are also able to bond with soft tissues (Hench 2006). The potential application of bioactive glasses for wound healing has been reviewed by Naseri et al. (2017). In general, the advantages of bioactive glasses include bonding to both hard and soft tissues, biodegradability, improving adhesion, as well as proliferation and differentiation of cells, high compositional flexibility due to utilizing useful cations for tissue restoration, and angiogenesis (Hench 2006; Rahaman et al. 2011). The capability of using various ions in the composition of bioglasses gives them some favorable properties such as antibacterial, blood coagulation, and healing acceleration (Bellantone et al. 2002; Ostomel et al. 2006; Miola and Verné 2016). In addition to the above-mentioned advantages, bioactive glasses can improve angiogenesis. One requirement for successful application of engineered scaffold is the development of new blood vessels, i.e., angiogenesis. If neovascularization does not occur in a three-dimensional scaffold, the viability of cells would be restricted due to lack of oxygen and nutrients. One solution is to approach using growth factors, although application of these proteins is not possible because they are destroyed under the temperature or chemical solutions, which are inevitable parts of scaffold fabrication. Therefore, angiogenesis is considered as one of the most important properties of bioactive glasses.

A 64S bioactive glass is a silicate-based glass with a composition of 64 SiO_2_-31 CaO-5 P_2_O_5_ (mol%). Bioactivity and biological properties of this glass have been investigated in some research works (Saboori et al. 2009; Imani Fooladi et al. 2013). Divalent oxides such as ZnO, SrO, and MgO can also be substituted partially with CaO to improve the biological properties of the glass (Balamurugan et al. 2007; Gentleman et al. 2010). For example, MgO has been reported to be effective in angiogenesis by inducing nitric oxide production in endothelial cells (Bose et al. 2013). A comprehensive review on Mg-containing bioactive glasses for biomedical application has been published by Diba et al. (2012). Also, it has been indicated that CoO can improve angiogenesis by inducing hypoxia condition. Hypoxia can activate hypoxia-inducible factor 1 (HIF-1), which is an important factor for the development of angiogenesis (Bose et al. 2013).

Despite these interesting and useful properties, the use of bioactive glasses is restricted due to their brittleness. To solve this problem, the combination of BG with biopolymers could be beneficial. In our previous work, we synthesized and investigated the properties of collagen-coated poly(lactic-*co*-glycolic acid) (PLGA) fibrous scaffold for skin tissue engineering applications (Sadeghi et al. 2016). Collagen is the main structural protein in the extracellular matrix which is biocompatible, biodegradable, and has shown wound-healing properties (Li et al. 2002; Rho et al. 2006; Chen et al. 2008; POWELL et al. 2008). PLGA is a biodegradable poly(α-hydroxyester), which possesses better mechanical properties than natural polymers such as collagen (Dhandayuthapani et al. 2011). In the present study, we aim to add BG to the above-mentioned scaffold to improve its biological properties.

Boccaccini and Maquet fabricated PLGA/Bioglass^®^ by thermally induced phase separation (TIPS) method and with different percentages of bioactive glass at maximum 50 wt%. They showed that the addition of BG to the scaffold increases water absorption and has a buffering effect, which may prevent inflammatory response toward acidic degradation of PLGA (Boccaccini 2003). Blaker et al. fabricated PDLLA and PDLLA/Bioglass^®^ by TIPS method and showed better bioactivity, viability, and attachment of osteoblast cells to the composite scaffold compared to PDLLA alone (Blaker et al. 2003). Liverani et al. incorporated 30 wt% of BG with respect to PCL weight percentage in electrospun PCL/chitosan scaffold (Liverani et al. 2018). They detected no HCA peak in XRD analysis after 7 days of immersion in SBF solution. Composites of polypeptide poly(N3-Cbz-l-lysine) (PZL)/PLGA with sol–gel-derived 45S5 bioglass were synthesized using negative NaCl-templating method (Cui et al. 2016). In vitro tests indicated that the addition of BG enhances adhesion, spreading, and proliferation of MC3T3-E1 cells, as well as inducing MC3T3-E1 differentiation to osteoblasts cells. Also the incorporation of sol–gel-derived BG into PLGA scaffold caused osteo-inductive properties and improved mechanical properties after incubation in SBF (Filipowska et al. 2014). Significant increment in the secretion of vascular endothelial growth factor (VEGF) from CCD-18Co myofibroblast (Keshaw et al. 2009) and L929 fibroblast (Day et al. 2005) has been reported for PLGA containing 45S5 Bioglass^®^ compared to a neat PLGA. Moreover, it has been observed that this effect (i.e., stimulation of angiogenic growth factors) is dose dependent, and high concentration of BG has a negative effect on growth factor secretion. Besides biological properties, surface coating of electrospun PLGA fibers with mesoporous bioactive glass improved the capability of scaffold for BMP-2 delivery (Li et al. 2015). Addition of BG to collagen has been studied in some research. Enhanced proliferation of human microvascular endothelial cells (HMVEC) and greater VEGF mRNA production was observed by the addition of specific amounts of glass to the collagen sponge (Leu and Leach 2008). Nanosized bioactive glass (nBG) with particle size in the range of 20–30 nm was added to bovine type I collagen film; composites containing 10 wt% nBG enhanced angiogenesis, while its 20 wt% hindered this property (Vargas et al. 2013). Wheeler et al. compared elastin-like polypeptides (ELP)-collagen and ELP-bioglass-collagen composites; they found that mechanical properties and ALP activity increased by the addition of bioglass (Wheeler et al. 2013).

Although the bioactive glass addition to collagen and PLGA has been studied separately, to the best of our knowledge, no research has investigated the effect of bioactive glass addition to the PLGA/collagen composite scaffold. Therefore, in this study, we are conducting a set of experiments in vitro to observe the effects of Co- or Mg-doped 64S bioactive glass on biological properties of PLGA/collagen fibrous scaffold.

## Materials and methods

### Materials

MgO- and CoO-doped 64S bioactive glasses with particle size of 20–50 nm were acquired in the Baqiyatallah Research Center (Tehran, Iran). The bioactive glasses were synthesized by the sol–gel method according to the literature (Imani Fooladi et al. 2013). The compositions of the two type bioactive glasses are shown in Table 1. MTT (3-[4,5-dimethylthiazol-2-yl]-2,5 diphenyltetrazolium bromide) and phosphate-buffered saline (PBS) were purchased from Sigma-Aldrich. Dimethylsulfoxide (DMSO), Dulbecco’s modified Eagle medium (DMEM), and fetal bovine serum (FBS) were prepared from Invitrogen (Germany).

**Table 1:** Compositions of bioactive glasses in mol%

	SiO_2_	CaO	P_2_O_5_	MgO	CoO
Mg-doped BG	64	26	5	5	–
Co-doped BG	64	26	5	–	5

### Fabrication of coated scaffold

Collagen-coated PLGA electrospun fibers were produced with a thickness of approximately 0.2 mm according to the authors’ previous work (Sadeghi et al. 2016). In brief, 20 w/v% of PLGA was dissolved in DMF/THF with a ratio of 1:3 and stirred for 12 h. Fabrication of scaffold was conducted by electrospinning machine (ANSTCO-RN/I, Iran). The obtained PLGA mats were hydrolyzed with 0.1 N concentration of NaOH to produce hydroxyl and carboxyl groups on the fibers’ surface. The activated fibers were immersed in a 2 mg/mL collagen solution in acetic acid (%0.5 by volume) for 5 h at 4 °C temperature. The cross-linking process was carried out using EDC/NHS (4/1) in 0.05 M MES.

PLGA/collagen samples were cut in 12-well plate size and sterilized on both sides. For coating with bioactive glass, slurry of 0.1 (w/v) BG nanoparticles was prepared in distilled water. After ultrasonication for 30 min, each slurry sample was poured into the 12-well polystyrene tissue culture plates. The plates were air dried in a laminar air hood. Different samples, including PLGA/collagen scaffold without bioactive glass, scaffold coated with Mg-doped bioactive glass, and scaffold coated with Co-doped bioactive glass were named uncoated, BG-Mg, and BG-Co, respectively.

### Characterization

All characterizations were carried out on PLGA/collagen-uncoated scaffold and 64S bioglass-coated scaffolds (BG-Mg and BG-Co).

#### MTT assay

The disc-shaped scaffolds with a diameter of 20 mm were sterilized by UV for 20 min. After soaking in sterile PBS (pH 7.4) for 2 h, the samples were placed in a 12-well plate in triplicate and 3T3 fibroblast cell lines were seeded into the 12-well cell culture plates (2 × 10^4^ cells/well). The suspensions were cultured in DMEM with 10% FBS and 100 U mL^−1^ penicillin/100 μgmL^−1^ streptomycin, and incubated in 5% CO_2_ at 37 °C. Cell proliferation was assessed after 24, 48, and 72 h by the addition of MTT solution to culture medium. After incubation for 2 h, MTT reaction medium was removed and 550 μL DMSO was added to each well. Elisa plate reader (ELX808, Biotek) measured the absorbance at 570 nm. The cell viability was calculated after subtraction of OD from the scaffolds without cell seeding.

#### Cell adhesion

Circular discs of scaffolds coated with CoO or MgO 64S bioglasses were prepared, sterilized, and seeded in triplicates with 3T3 fibroblast cells (2 × 10^4^ cells/ml) and cultured in a 12-well plate as mentioned above. The plates were incubated for 3 days in 5% CO_2_ at 37 °C. After incubation, the culture plates containing scaffolds were washed three times with PBS to remove the unattached cells. Attached cells were fixed in 2.5% glutaraldehyde solution for 3 h. Thereafter, the scaffolds were rinsed twice in distilled water and dehydrated with high concentrations of ethanol for 15 min. After drying in a desiccator, the samples were sputter coated with gold and evaluated by scanning electron microscope (LEO 1450VP).

#### Angiogenesis evaluation

The amount of VEGF secreted by 3T3 fibroblast cells was assessed using supernatant of cell culture after 24, 48, and 72 h. The measurements were carried out by quantitative enzyme-linked immunosorbent assay (ELISA) according to the manufacturer’s instructions (Mouse VEGF Quantikine ELISA Kit; R&D Systems, UK). The optical density of each well was determined using a microplate reader at a wavelength of 450 nm. The results are presented in pg/mL of VEGF. The values for secreted VEGF are expressed after subtraction of the amount of VEGF measured in culture medium plus 10% FBS without cells.

#### ICP-OES

To determine the concentration of released ions in culture medium, supernatants in three different durations (24, 48, and 72 h) were collected with three replications. The samples were diluted by 10% (v/v) nitric acid; the supernatants to nitric acid ratio were 1:10. Ion concentrations of Ca, Co, Mg, P, and Si were measured by inductively coupled plasma-atomic emission spectroscopy (ICP-OES; Spectro Arcos, Germany).

### Statistical analysis

Data from MTT, VEGF, and ICP-OES tests were represented as mean ± standard deviation and statistically analyzed by one-way analysis of variance (ANOVA). *p* value < 0.05 was set as statistically significant.

## Results

### Microstructure and porosity of PLGA/collagen scaffold

**Fig. 1:**
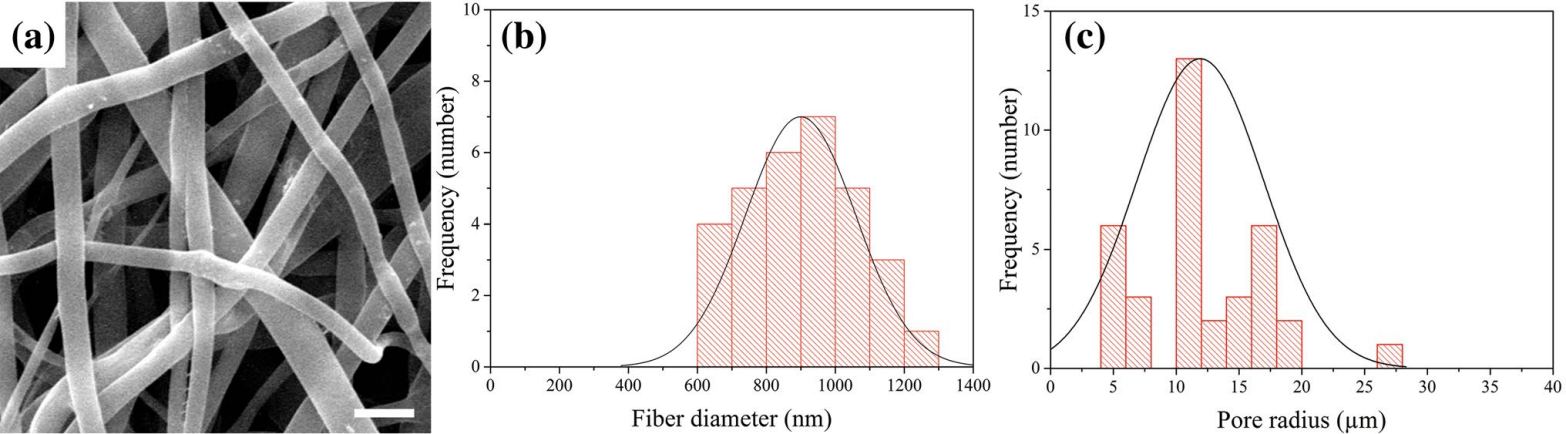
**a** SEM image of scaffold comprising collagen-coated PLGA electrospun fibers; the scale bar shows 2 µm. **b** Histogram diagram of fiber diameter and **c** pore size distribution of the scaffold

Figure 1 depicts microstructure, fiber diameter, and pore diameter frequency distribution histograms of PLGA/collagen scaffold. The SEM image shows beadles and approximately uniform fibrous scaffold. The fiber diameter lies in the range of 600–1300 nm, and the average diameter of fibers is 965 nm. In addition, the result of mercury porosimetry demonstrates that the pore diameter is in the range of 4–30 m with an average of 11.3 m.

### Cell attachment

**Fig. 2:**
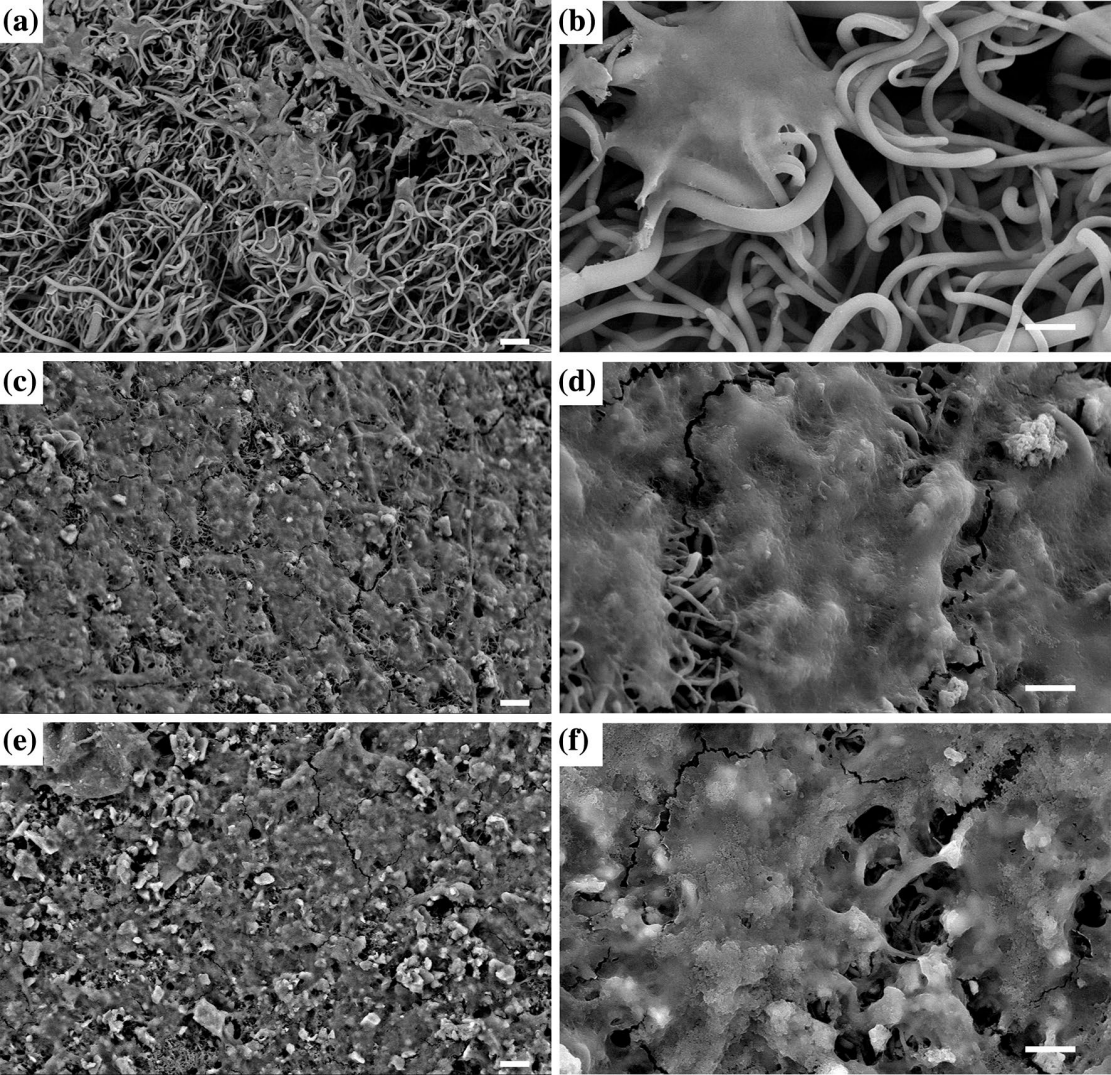
SEM images with two magnifications for fibroblast attachment to: **a**, **b** PLGA/collagen scaffold; **c**, **d** scaffold coated with Mg-doped bioactive glass (BG-Mg); and **e**, **f** scaffold coated with Co-doped bioactive glass (BG-Co). The scale bars for images **a**, **c**, and **e** represent 20 µm and for images **b**, **d**, and **f** represent 5 µm

Fibroblast attachment after 3 days is shown in Fig. 2a–f for BG-coated and uncoated scaffolds. SEM images show that BG nanoparticles have been agglomerated during the coating process and they range from nanometer to micrometer. It is obvious that more cells have attached on the scaffolds coated with bioactive glasses.

### MTT assay

**Fig. 3:**
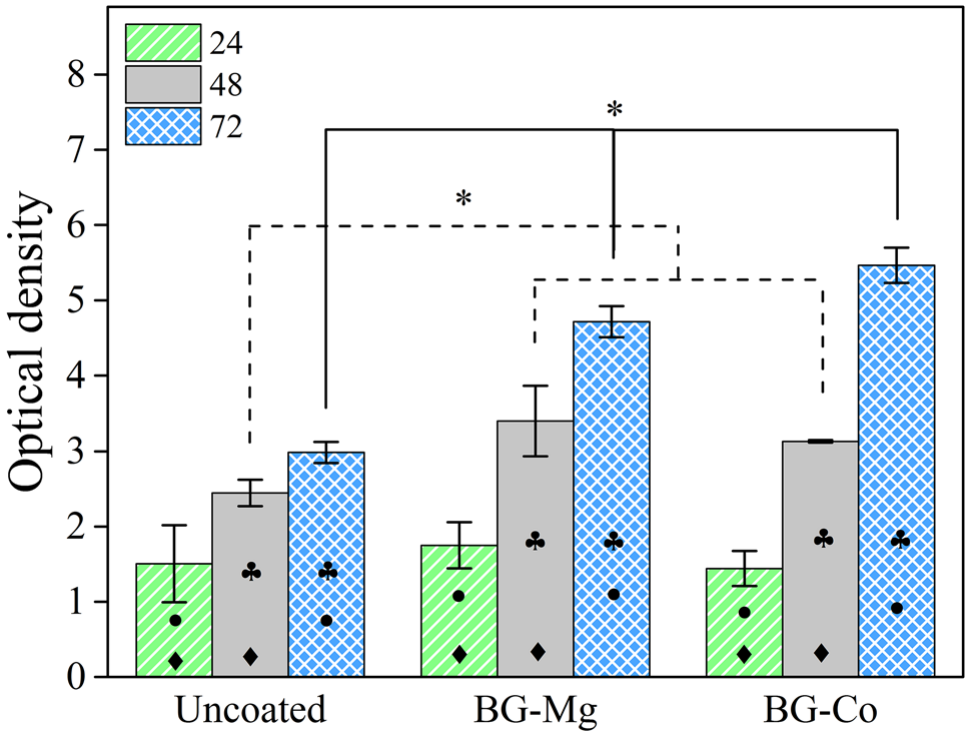
MTT assay results for uncoated, BG-Mg, and BG-Co samples. Black-filled diamond indicates significant difference (*p *< 0.05) of cell viability between 24 and 48 h. Black-filled club indicates significant difference (*p *< 0.05) of cell viability between 48 and 72 h. Black-filled circle indicates significant difference (*p* < 0.05) of cell viability between 24 and 72 h. Asterisk indicates significant difference (*p* < 0.05) of cell viability between different samples

Figure 3 shows the results of MTT assay for PLGA/collagen-uncoated scaffold and the bioactive glass-coated scaffolds, i.e., BG-Co and BG-Mg. The viability of cells increased for all samples during 3 days. Also, no significant difference in the viability of cells can be observed between uncoated and BG-coated samples at 24 h of fibroblast culturing. However, after 48 and 72 h, it is clear that the OD absorbance for both BG-Co and BG-Mg samples is significantly higher than uncoated sample. There is not any difference in OD between BG-Mg and BG-Co except on the third day; BG-Co shows a higher population of viable fibroblast cells after 72 h.

### VEGF secretion

**Fig. 4:**
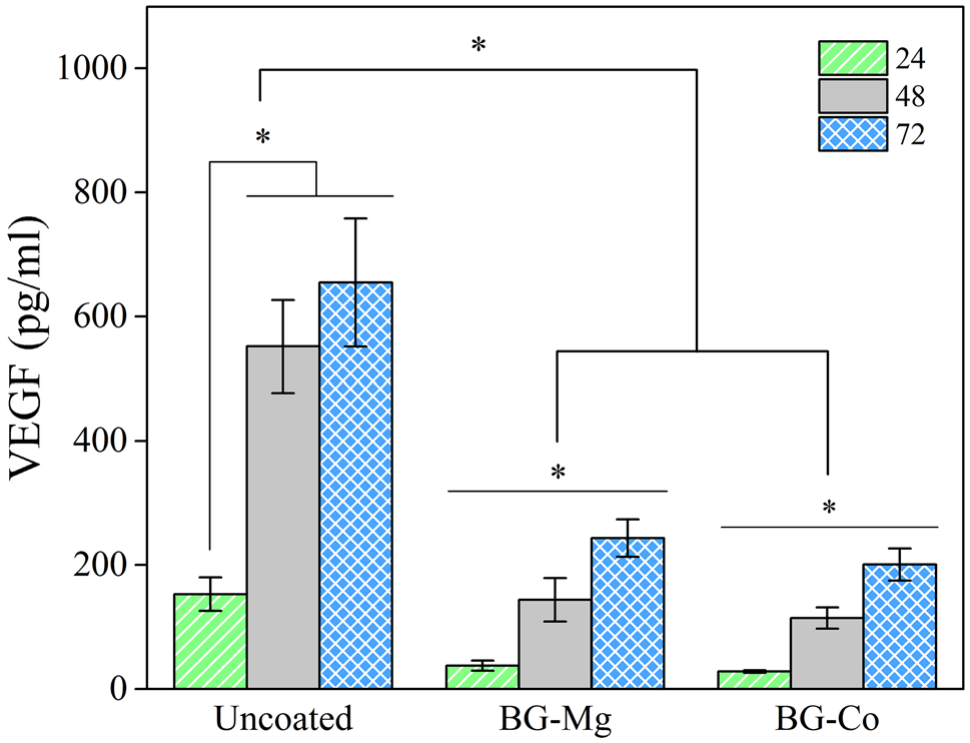
Measurement of VEGF secretion by 3T3 fibroblast cells after 24, 48, and 72 h of cell culturing for uncoated, BG-Mg, and BG-Co samples. The asterisk indicates significant level (*p *< 0.05) of VEGF secretion

Figure 4 represents secreted vascular endothelial growth factor from fibroblast cells. The amount of VEGF in all samples has increased significantly by increasing the time. It can be seen that the amount of VEGF secretion of uncoated sample is much higher than BG-coated scaffolds during different periods of the experiment. Comparing BG-Co sample with that of BG-Mg, no significant difference can be recorded.

### Ion release measurement

**Fig. 5:**
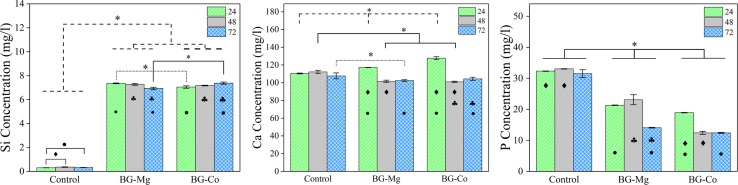
ICP-OES results showing concentrations of Si, Ca, and P for uncoated, BG-Mg, and BG-Co samples. Data were collected after 24, 48, and 72 h of soaking samples in culture medium. Black-filled diamond indicates significant difference (*p* < 0.05) of ion concentration between 24 and 48 h. Black-filled club indicates significant difference (*p* < 0.05) of ion concentration between 48 and 72 h. Black-filled circle indicates significant difference (*p* < 0.05) of ion concentration between 24 and 72 h. Asterisk indicates significant difference (*p* < 0.05) of cell viability between different samples

**Fig. 6:**
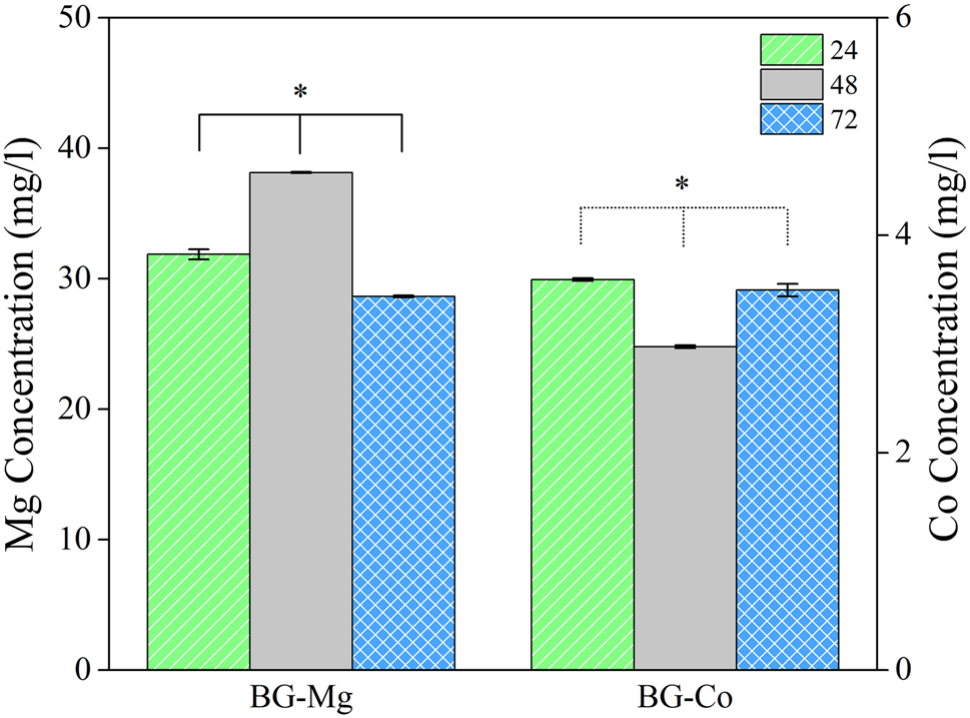
Ion concentrations of Mg and Co, respectively, for BG-Mg and BG-Co samples. Data were collected after 24, 48, and 72 h of samples soaking in culture medium

Si, Ca, and P concentrations in culture medium for 24, 48, and 72 h are given in Fig. 5a–c. The concentration of Si for uncoated sample is far less than that of BG-coated samples. Conversely, the concentration of phosphorus for uncoated sample is higher than BG-coated samples at all 3 days. In the case of Ca, the uncoated sample has the lowest concentration compared to BG-coated samples on day 1, but it has the highest concentration on days 2 and 3. Figure 6 shows magnesium and cobalt concentrations at different times for BG-Mg and BG-Co samples, respectively. The concentration of Mg increased at day 2, but then decreased, while the concentration of Co decreased at second day and then started increasing.

## Discussion

The microstructure of a scaffold, especially pore structure and fiber diameter, has a very important influence on cell adhesion, proliferation, migration, and differentiation. For cell nutrition, scaffolds should possess a porous structure with interconnected pores. The diameter of pores should not be too small to restrict the migration of cells into the pores and it should not be too large to limit cell adhesion due to large bridging distances across the pores (Lowery et al. 2010). Also, fiber diameter influences attachment, spreading morphology, and proliferation of cells (Kumbar et al. 2008; Hodgkinson et al. 2014; Li et al. 2016). The obtained fiber diameter for the synthesized PLGA/collagen scaffold (Fig. 1b) is in good agreement with Kumbar et al. findings, which showed that the best fiber diameter for fibroblast attachment to PLGA scaffold was in the range of 350–1100 nm (Kumbar et al. 2008). Figure 2a, b illustrates well-spread and flattened morphology of the attached fibroblast cells on PLGA/collagen scaffold. However, the addition of BG has improved cell attachment to PLGA/collagen scaffold. As it is observed in Fig. 2c–f, the amount of attached cells has increased impressively and more area of the scaffold is covered by fibroblast cells. It could be due to nanoscale topology of the bioactive glass and increased surface area of the scaffold, which provides better situation for cell attachment (Teixeira et al. 2004; Wang et al. 2013).

Cell attachment in turn can influence proliferation and differentiation. Figure 3 indicates that better proliferation has occurred for glass-containing scaffolds. This could be due to surface chemistry and topography. Nanotopography and nanoscale pores can improve cell proliferation (Zheng et al. 2009). Beside surface characteristics, bioactive glass can release ions which stimulate proliferation-associated signaling pathways (Hench 2009; Hoppe et al. 2011).

It has been shown that the small amount of bioactive glass enhances growth factor secretion. Gorustovich et al. have provided a comprehensive review of the effect of BG on angiogenesis (Gorustovich et al. 2010). Therefore, it is expected that the release of BG would enhance VEGF secretion from fibroblast cells for BG-containing scaffolds. Figure 4 shows that the amount of secreted VEGF is not significantly different for BG-Co and BG-Mg samples, but this value is higher for scaffold without bioactive glass. Day et al. have shown that 0.1% (w/v) of micron-sized BG is suitable for increasing VEGF secretion and higher concentrations would diminish it (Day et al. 2005). In the present research, this quantity of BG has a negative effect on VEGF amount. This could be due to nanometer size and high surface area of BG particles, which promotes fast degradation and increases ionic concentration. Moreover, the bioactive glass methodical synthesis, i.e., sol–gel, makes the surface more porous compared to the melt-derived glass and, therefore, it makes it more prone to fast degradation (Sepulveda et al. 2002). Comparing uncoated sample with BG-coated samples, it is obvious that in the presence of bioactive glasses, Si concentration in culture medium has increased significantly (Fig. 5a). This is an indication of glass degradation. Moreover, it can be observed that almost all Si concentrations have incremented by the first 24 h; in other words, bioactive glasses have degraded very fast. It seems that inhibition of VEGF secretion in BG-containing scaffolds is due to the fast and uncontrolled release of ions. Therefore, to improve angiogenesis and cell adhesion simultaneously, optimum particle size of bioactive glass should be determined. Figure 5 shows the ionic concentrations of Si, Ca, and P concentration in culture medium. High concentrations of Ca, P, and Mg ions for uncoated sample are due to inorganic salt constituents of DMEM including CaCl_2_, NaH_2_PO_4_, and MgSO_4_. During the first day, Ca^2+^ concentration has increased in BG-containing samples, which is due to glass degradation (Fig. 5b). However, the concentration is decreased in the following days. The reduction of ion concentration in the presence of bioactive glass is more obvious for phosphorus concentration (Fig. 5c). These observations show that Ca and P ions have been adsorbed on the surface of BG-containing scaffolds. As indicated by Hench and other researchers (Hench 1993; Hayakawa et al. 1999; Lin et al. 2005), the degradation of bioactive glass produces a hydrated silica gel layer on the surface, which provides a suitable place for Ca^2+^ and PO_4_^−3^ deposition. The reduction of P concentration in culture medium for both 45S5 and 58S bioactive glasses has been reported by Sepulveda et al. (Sepulveda et al. 2002). Therefore, the reduction of Ca and P ion concentrations in the culture medium is attributed to the formation of a superficial gel layer on bioactive glass and its ionic adsorption characteristics.

Figure 6 shows magnesium and cobalt concentration for BG-Mg and BG-Co, respectively. Co ion is not available in DMEM and so the only source of cobalt ion is Co-doped bioactive glass. Data show that the concentration of Co ion is in its highest amount in the first day after soaking in DMEM. This is another indication of fast degradation of the bioactive glass. The Co concentration is decreased in the second day and then increased. However, the magnesium ion concentration is increased until the second day and then it is decreased. The alterations in Co and Mg concentrations in different days show the unstable surface adsorption behavior of bioactive glass in the culture medium.

Also, the addition of bioactive glass has reduced contraction of the PLGA/collagen scaffold in contact with the culture medium (data not shown). Many studies have reported shrinkage of electrospun polymeric scaffolds when they are exposed to culture medium (Xie et al. 2011; Cui et al. 2012). It happens because during electrospinning process, polymer chains are exposed to a high electric field and stretched along field direction. This produces inner stress in the elongated fibers. When polymer is soaked in solution with high enough temperature, i.e., close or higher than polymer glass transition temperature, macromolecules acquire mobility and consequently fibers are shrunk (Ru et al. 2015). For successful implantation of skin substitutes in skin surgeries, no size alteration should happen. Moreover, dimensional instability and porosity changes of scaffold can negatively affect cell attachment and infiltration into the scaffold (Ru et al. 2015). Therefore, reducing the contraction of scaffold is another positive effect of bioactive glass addition.

## Conclusion

We investigated the effect of CoO- or MgO-doped 64S bioglass nanoparticles coating on PLGA/collagen composite scaffold. The analysis showed fast degradation of bioglass in culture medium. Although the concentration of Si and Co increased due to bioglass degradation, the concentrations of P and Ca decreased after 3 days, which was attributed to ion precipitation on bioactive glass nanoparticles. Coating of PLGA/collagen with BG nanoparticles significantly increased attachment and viability of the fibroblast cells and enhanced dimensional stability of the scaffold. However, the VEGF secretion decreased in BG-coated sample compared to the uncoated scaffold. This could be attributed to a high concentration of ions in culture medium because of nanoparticle fast degradation. Future works should determine the optimum concentration/particle size of bioactive glass nanoparticles for improving angiogenesis of PLGA/collagen scaffold.

## Electronic supplementary material

Below is the link to the electronic supplementary material.

Supplementary material 1 (DOCX 806 kb)
