# Supplementary material for: Effect of bioactive glass nanoparticles on biological properties of PLGA/collagen scaffold
Source: Prog Biomater. 2018 May 11;7:111–9. doi: 10.1007/s40204-018-0089-y (PMC6068071; doi:10.1007/s40204-018-0089-y)

**Fig S1** SEM image of cultured scaffold containing bioactive glass and EDX spectra of the specified area in the SEM image.


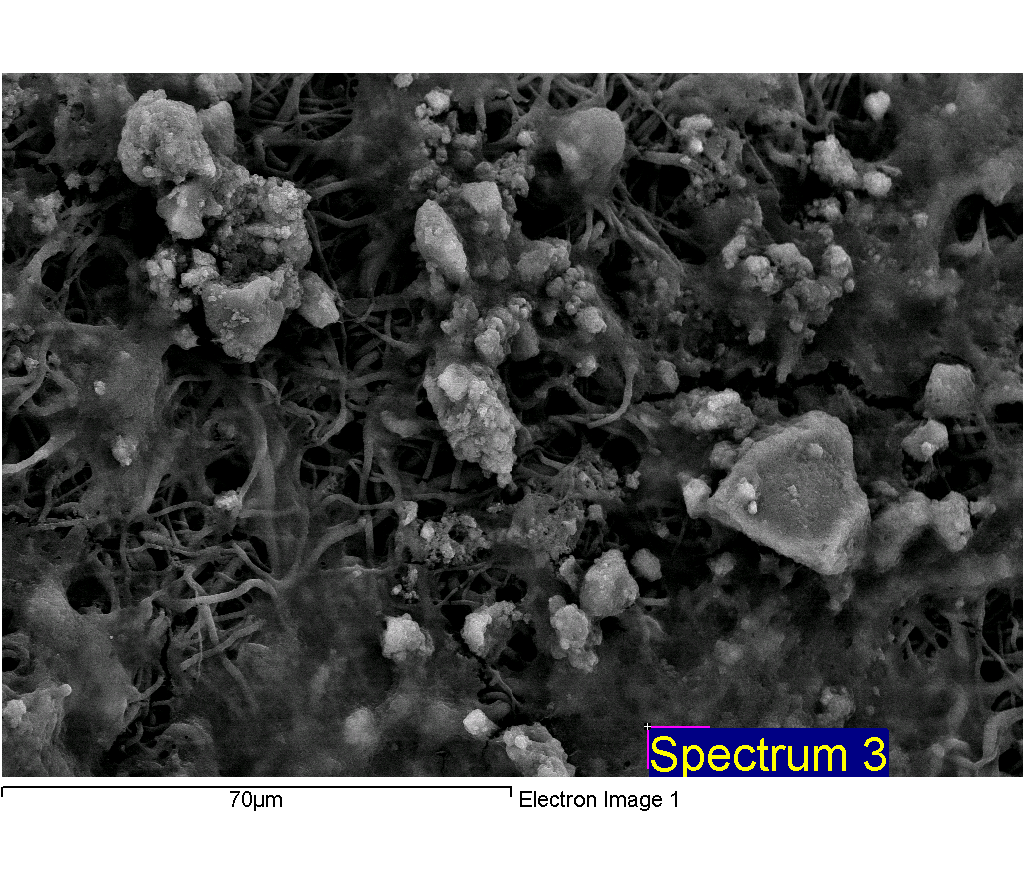

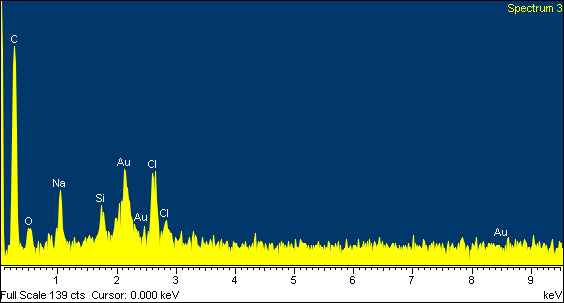


**Fig S2** SEM image of cultured scaffold containing bioactive glass and EDX spectra of the specified area in the SEM image.
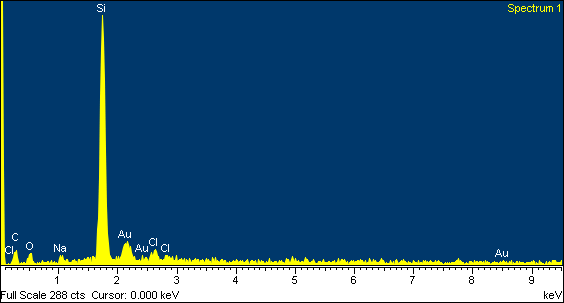

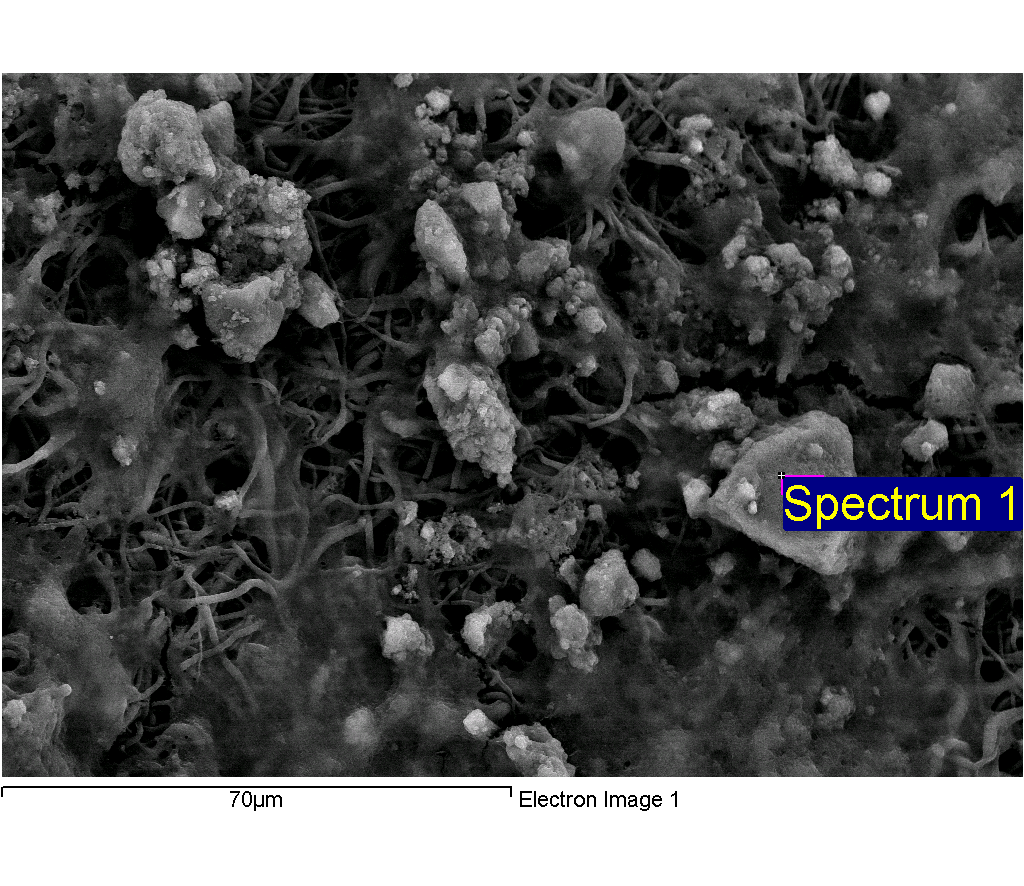

Supplement: Supplementary file 1 — Supplementary material 1 (DOCX 806 kb) [file 40204_2018_89_MOESM1_ESM.docx]
